# 4-Fluoro-2-(phenyl­amino)­benzoic acid

**DOI:** 10.1107/S2414314624001974

**Published:** 2024-03-06

**Authors:** Jingxi Li, Sihui Long

**Affiliations:** a School of Chemical Engineering and Pharmacy, Wuhan Institute of Technology, Wuhan, Hubei 430205, People’s Republic of China; University of Aberdeen, United Kingdom

**Keywords:** *Z*′ = 2, twisted conformation, acid–acid dimer, crystal structure

## Abstract

The title compound, C_13_H_10_FNO_2_, was obtained by the reaction of 2-bromo-4-fluoro­benzoic acid with aniline. There are two independent mol­ecules, *A* and *B*, in the asymmetric unit, with slight conformational differences: the dihedral angles between the aromatic rings are 55.63 (5) and 52.65 (5)°. Both mol­ecules feature an intra­molecular N—H⋯O hydrogen bond. In the crystal, the mol­ecules are linked by pairwise O—H⋯O hydrogen bonds to form *A*–*B* acid–acid dimers and weak C—H⋯F inter­actions further connect the dimers.

## Abstract 2

There are two crystallographically independent mol­ecules in the asymmetric unit of the title compound, which are linked by pairwise O—H⋯O hydrogen bonds.

## Graphical abstract

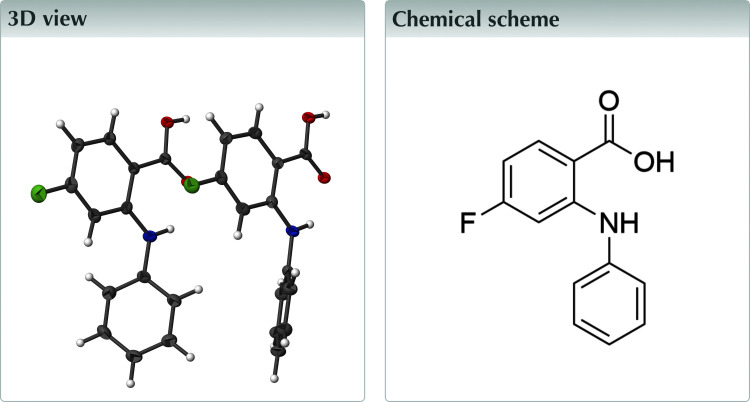

## Structure description

Non-steroidal anti-inflammatory drugs are among the most commonly utilized medicines globally (Abdu *et al.*, 2020 ▸). They exhibit anti-inflammatory, anti­pyretic, and analgesic properties. They are available as both prescription and over-the-counter medications and are employed to treat fever, acute or chronic pain, and various inflammatory conditions such as osteoarthritis and rheumatoid arthritis (Brennan *et al.*, 2021 ▸). Anthranilic acid derivatives represent a crucial subset of non-steroidal anti-inflammatory drugs.

**Figure 1:**
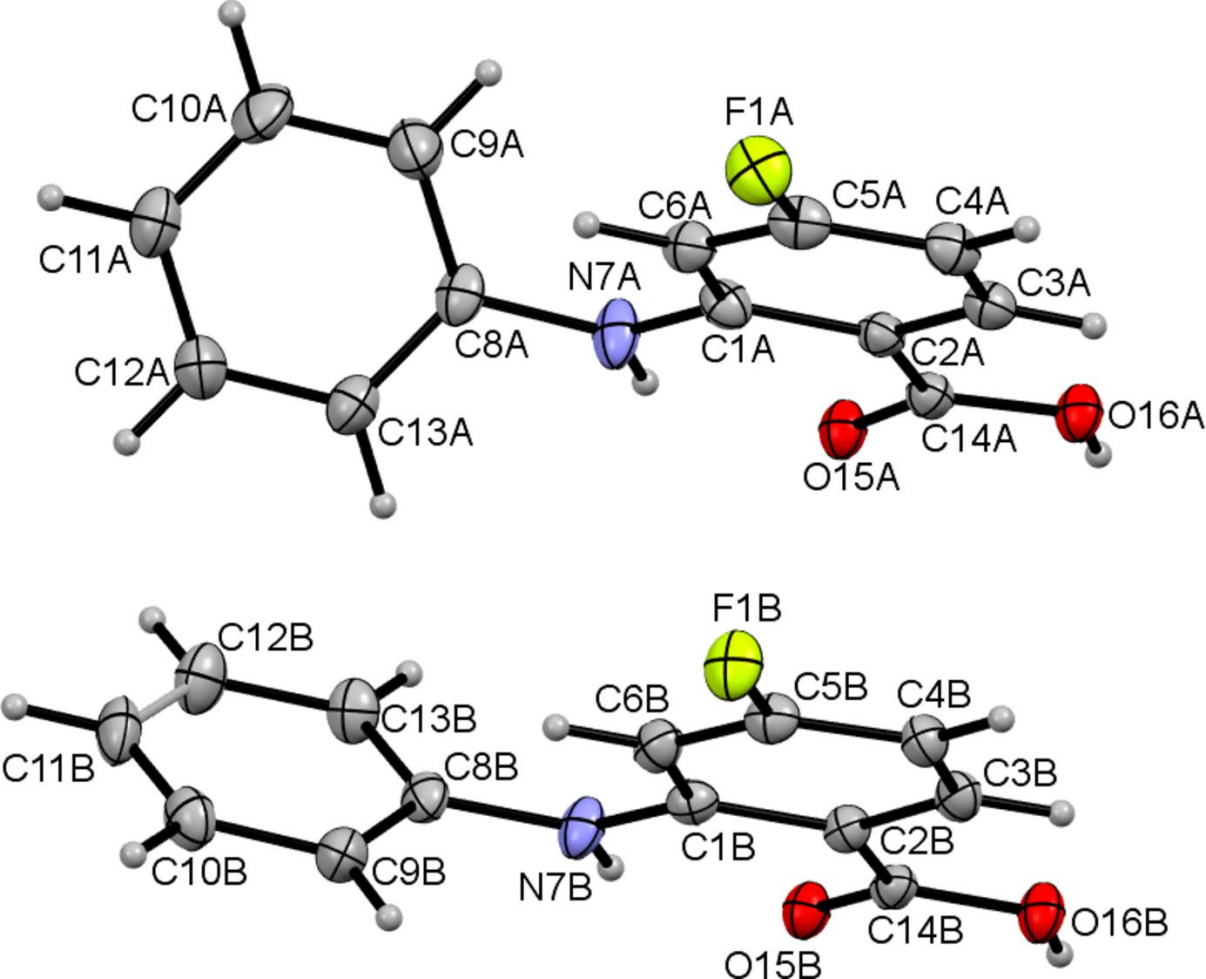
The mol­ecular structure of the title compound with displacement ellipsoids drawn at the 50% probability level.

**Figure 2:**
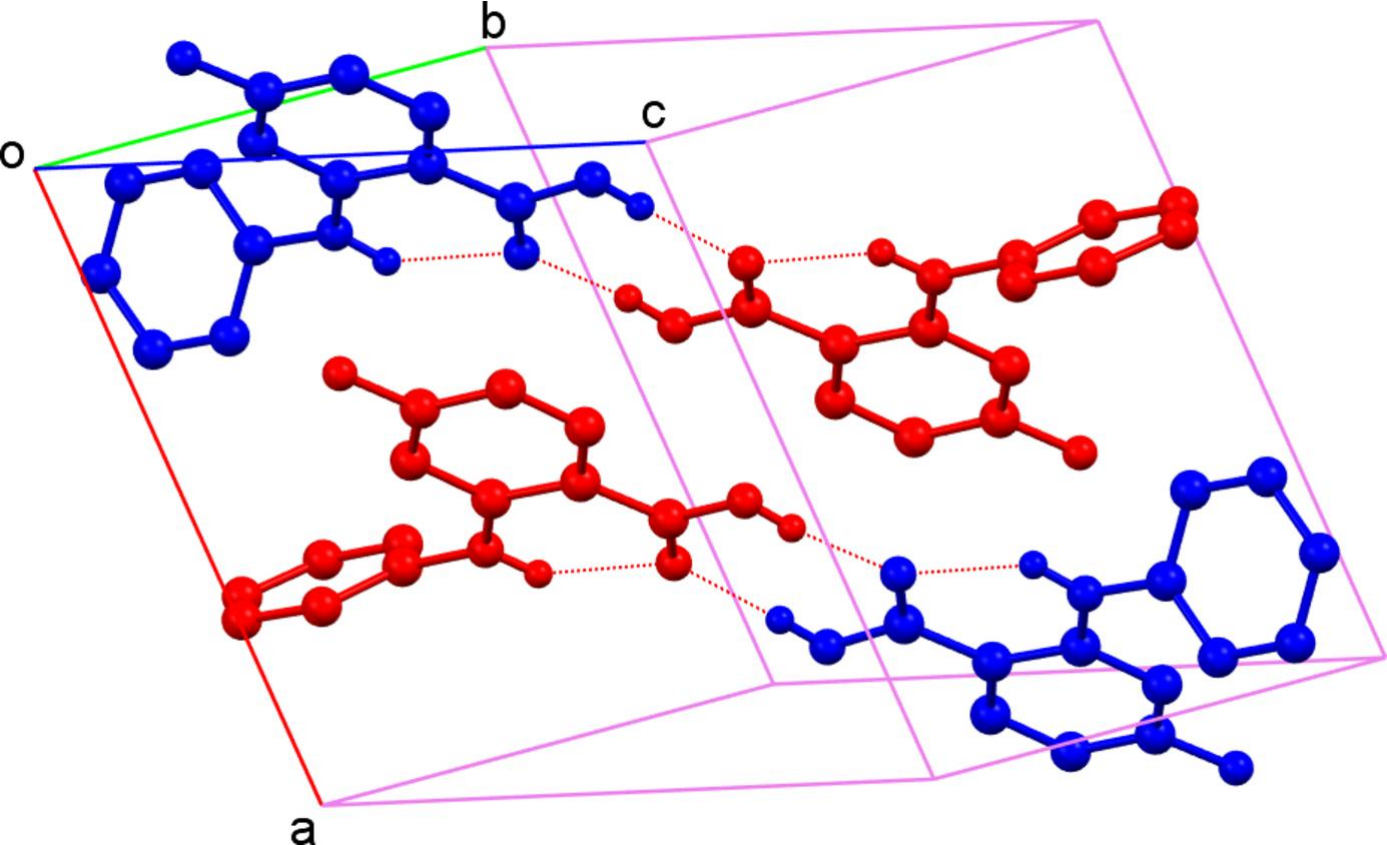
Packing of the mol­ecules in the crystal (for clarity, H atoms not involved in hydrogen bonding are omitted). The C1*A* mol­ecule is shown in red and the C1*B* mol­ecule in blue.

The title compound (Fig. 1 ▸), an anthranilic acid derivative, was synthesized employing the Ullmann reaction (Sambiagio *et al.*, 2014 ▸). Crystallization from acetone solution led to suitable single crystals for structure determination by single-crystal X-ray diffraction, which revealed the asymmetric unit to consist of two mol­ecules, *A* (containing C1*A*) and *B* (containing C1*B*) (Fig. 1 ▸). The dihedral angles between the C1*A*–C6*A*/C8*A*–C13*A* and C1*B*–C6*B*/C8*B*–C13*B* aromatic rings are 55.63 (5) and 52.65 (5)°, respectively. Both mol­ecules feature an intra­molecular N—H⋯O hydrogen bond (Table 1 ▸). In the extended structure, the mol­ecules form *A*–*B* dimers by way of pairwise O—H⋯O hydrogen bonds (Fig. 2 ▸, Table 1 ▸). Two weak C—H⋯F inter­actions are also observed.

## Synthesis and crystallization

**Figure 3:**
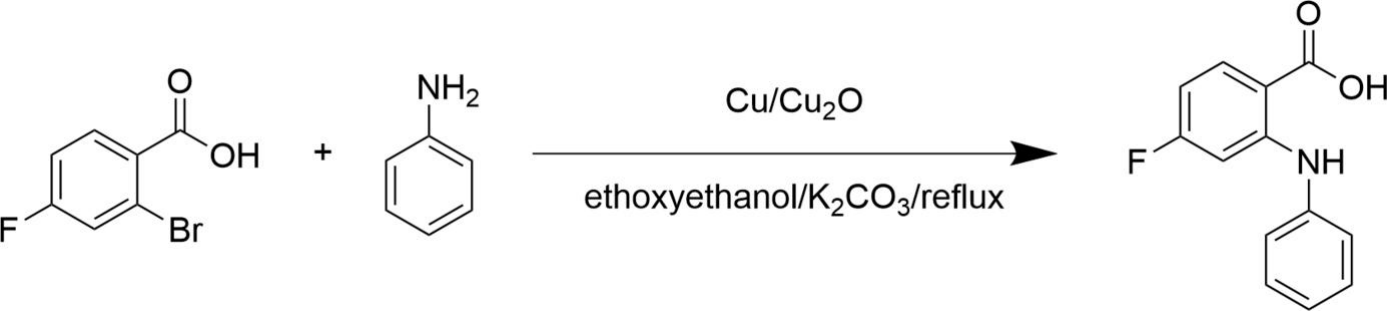
Synthesis of the title compound.

The title compound was prepared by reacting 2-bromo-4-fluoro­benzoic acid and aniline in the presence of a Cu catalyst at 403 K (Fig. 3 ▸). The product was purified by column chromatography. Single crystals were obtained by slowly evaporating an acetone solution of the title compound.

## Refinement

Crystal data, data collection and structure refinement details are summarized in Table 2 ▸.

## Supplementary Material

Crystal structure: contains datablock(s) global, I. DOI: 10.1107/S2414314624001974/hb4457sup1.cif

Structure factors: contains datablock(s) I. DOI: 10.1107/S2414314624001974/hb4457Isup2.hkl

Supporting information file. DOI: 10.1107/S2414314624001974/hb4457Isup3.cml

CCDC reference: 2336149

Additional supporting information:  crystallographic information; 3D view; checkCIF report

## Figures and Tables

**Table 1:** Hydrogen-bond geometry (Å, °)

*D*—H⋯*A*	*D*—H	H⋯*A*	*D*⋯*A*	*D*—H⋯*A*
N7*A*—H7*A*⋯O15*A*	0.88	1.98	2.673 (2)	135
N7*B*—H7*B*⋯O15*B*	0.88	1.98	2.6770 (19)	136
O16*A*—H16*A*⋯O15*B* ^i^	0.84	1.80	2.6413 (19)	176
O16*B*—H16*B*⋯O15*A* ^i^	0.84	1.77	2.6099 (19)	174
C4*A*—H4*A*⋯F1*B* ^ii^	0.95	2.48	3.426 (2)	174
C4*B*—H4*B*⋯F1*A* ^ii^	0.95	2.52	3.464 (2)	173

Symmetry codes: (i) 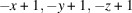 ; (ii) 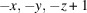 .

**Table 2:** Experimental details

Crystal data	
Chemical formula	C_13_H_10_FNO_2_
*M*_r_	231.22
Crystal system, space group	Triclinic, *P* 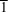
Temperature (K)	90
*a*, *b*, *c* (Å)	9.9550 (3), 10.0060 (3), 11.3320 (4)
α, β, γ (°)	89.4150 (14), 78.0130 (14), 78.9010 (14)
*V* (Å^3^)	1082.99 (6)
*Z*	4
Radiation type	Mo *K*α
μ (mm^−1^)	0.11
Crystal size (mm)	0.40 × 0.10 × 0.10

Data collection	
Diffractometer	Nonius KappaCCD diffractometer
Absorption correction	Multi-scan (*SCALEPACK*; Otwinowski & Minor, 1997 ▸)
*T*_min_, *T*_max_	0.958, 0.989
No. of measured, independent and observed [*I* > 2σ(*I*)] reflections	9760, 4918, 2625
*R*_int_	0.062
(sin θ/λ)_max_ (Å^−1^)	0.647

Refinement	
*R*[*F* ^2^ > 2σ(*F* ^2^)], *wR*(*F* ^2^), *S*	0.050, 0.131, 0.94
No. of reflections	4918
No. of parameters	309
H-atom treatment	H-atom parameters constrained
Δρ_max_, Δρ_min_ (e Å^−3^)	0.28, −0.29

Computer programs: *COLLECT* (Nonius, 2002 ▸), *DENZO-SMN* (Otwinowski & Minor, 1997 ▸), *SHELXS97* and *SHELXL97* (Sheldrick, 2015 ▸) and *XP* in *SHELXTL* (Sheldrick, 2008 ▸).

## full crystallographic data

### Crystal data

**Table:**

C_13_H_10_FNO_2_	*Z* = 4
*M**_r_* = 231.22	*F*(000) = 480
Triclinic, *P*1	*D*_x_ = 1.418 Mg m^−^^3^
Hall symbol: -P 1	Mo *K*α radiation, λ = 0.71073 Å
*a* = 9.9550 (3) Å	Cell parameters from 4887 reflections
*b* = 10.0060 (3) Å	θ = 1.0–27.5°
*c* = 11.3320 (4) Å	µ = 0.11 mm^−^^1^
α = 89.4150 (14)°	*T* = 90 K
β = 78.0130 (14)°	Rod, colorless
γ = 78.9010 (14)°	0.40 × 0.10 × 0.10 mm
*V* = 1082.99 (6) Å^3^	

### Data collection

**Table:**

Nonius KappaCCD diffractometer	4918 independent reflections
Radiation source: fine-focus sealed tube	2625 reflections with *I* > 2σ(*I*)
Graphite monochromator	*R*_int_ = 0.062
Detector resolution: 18 pixels mm^-1^	θ_max_ = 27.4°, θ_min_ = 1.8°
ω scans at fixed χ=55°	*h* = −12→12
Absorption correction: multi-scan (Scalepack; Otwinowski & Minor, 1997)	*k* = −12→12
*T*_min_ = 0.958, *T*_max_ = 0.989	*l* = −14→14
9760 measured reflections	

### Refinement

**Table:**

Refinement on *F*^2^	Primary atom site location: structure-invariant direct methods
Least-squares matrix: full	Secondary atom site location: difference Fourier map
*R*[*F*^2^ > 2σ(*F*^2^)] = 0.050	Hydrogen site location: inferred from neighbouring sites
*wR*(*F*^2^) = 0.131	H-atom parameters constrained
*S* = 0.94	*w* = 1/[σ^2^(*F*_o_^2^) + (0.0609*P*)^2^] where *P* = (*F*_o_^2^ + 2*F*_c_^2^)/3
4918 reflections	(Δ/σ)_max_ = 0.001
309 parameters	Δρ_max_ = 0.28 e Å^−^^3^
0 restraints	Δρ_min_ = −0.29 e Å^−^^3^

### Special details

**Table:**

Geometry. All esds (except the esd in the dihedral angle between two l.s. planes) are estimated using the full covariance matrix. The cell esds are taken into account individually in the estimation of esds in distances, angles and torsion angles; correlations between esds in cell parameters are only used when they are defined by crystal symmetry. An approximate (isotropic) treatment of cell esds is used for estimating esds involving l.s. planes.
Refinement. Refinement of F^2^ against ALL reflections. The weighted R-factor wR and goodness of fit S are based on F^2^, conventional R-factors R are based on F, with F set to zero for negative F^2^. The threshold expression of F^2^ > 2sigma(F^2^) is used only for calculating R-factors(gt) etc. and is not relevant to the choice of reflections for refinement. R-factors based on F^2^ are statistically about twice as large as those based on F, and R- factors based on ALL data will be even larger. The positions of the H atoms attached to the N and O atoms were obtained from a difference Fourier map, which were then relocated to idealised locations. The C-bound H atoms were positioned geometrically with C—H = 0.95 Å and refined as riding atoms. The constraint U_iso_(H) = 1.2U_eq_(C,N) or 1.5U_eq_(O) was applied in all cases.

### Fractional atomic coordinates and isotropic or equivalent isotropic displacement parameters (Å^2^)

**Table:**

	*x*	*y*	*z*	*U*_iso_*/*U*_eq_	
F1B	0.33716 (12)	0.00814 (11)	0.33502 (9)	0.0289 (3)	
C1B	0.5768 (2)	0.24263 (18)	0.29666 (16)	0.0187 (4)	
C2B	0.5628 (2)	0.28222 (18)	0.41950 (15)	0.0180 (4)	
C3B	0.4705 (2)	0.22914 (18)	0.50929 (16)	0.0209 (5)	
H3B	0.4611	0.2575	0.5908	0.025*	
C4B	0.3924 (2)	0.13739 (19)	0.48453 (16)	0.0213 (5)	
H4B	0.3298	0.1019	0.5463	0.026*	
C5B	0.4108 (2)	0.09982 (19)	0.36367 (17)	0.0207 (5)	
C6B	0.4976 (2)	0.14842 (18)	0.27121 (16)	0.0214 (5)	
H6B	0.5047	0.1190	0.1903	0.026*	
N7B	0.66309 (17)	0.29688 (16)	0.20633 (13)	0.0233 (4)	
H7B	0.7129	0.3520	0.2288	0.028*	
C8B	0.6802 (2)	0.2731 (2)	0.08018 (16)	0.0216 (5)	
C9B	0.7158 (2)	0.1412 (2)	0.02896 (16)	0.0255 (5)	
H9B	0.7313	0.0645	0.0780	0.031*	
C10B	0.7281 (2)	0.1238 (2)	−0.09443 (17)	0.0281 (5)	
H10B	0.7496	0.0343	−0.1294	0.034*	
C11B	0.7098 (2)	0.2344 (2)	−0.16717 (17)	0.0299 (5)	
H11B	0.7185	0.2214	−0.2515	0.036*	
C12B	0.6787 (2)	0.3644 (2)	−0.11607 (17)	0.0305 (5)	
H12B	0.6678	0.4410	−0.1659	0.037*	
C13B	0.6633 (2)	0.3839 (2)	0.00710 (16)	0.0262 (5)	
H13B	0.6411	0.4736	0.0415	0.031*	
C14B	0.6411 (2)	0.38018 (19)	0.45451 (16)	0.0191 (4)	
O15B	0.71882 (13)	0.44023 (12)	0.38231 (10)	0.0216 (3)	
O16B	0.62152 (14)	0.40054 (13)	0.57300 (10)	0.0231 (3)	
H16B	0.6748	0.4510	0.5876	0.035*	
F1A	−0.15859 (12)	0.01065 (11)	0.31096 (10)	0.0312 (3)	
C1A	0.0766 (2)	0.24951 (19)	0.27856 (16)	0.0209 (5)	
C2A	0.06407 (19)	0.28389 (18)	0.40285 (16)	0.0185 (4)	
C3A	−0.0259 (2)	0.22559 (19)	0.49136 (17)	0.0211 (5)	
H3A	−0.0339	0.2495	0.5738	0.025*	
C4A	−0.1028 (2)	0.13494 (19)	0.46294 (17)	0.0231 (5)	
H4A	−0.1640	0.0964	0.5235	0.028*	
C5A	−0.0867 (2)	0.10265 (19)	0.34142 (17)	0.0229 (5)	
C6A	−0.0025 (2)	0.15615 (18)	0.25109 (17)	0.0225 (5)	
H6A	0.0030	0.1306	0.1694	0.027*	
N7A	0.16563 (18)	0.30209 (17)	0.18986 (13)	0.0272 (4)	
H7A	0.2212	0.3499	0.2136	0.033*	
C8A	0.1777 (2)	0.28748 (19)	0.06299 (16)	0.0233 (5)	
C9A	0.0611 (2)	0.3164 (2)	0.01172 (18)	0.0324 (5)	
H9A	−0.0292	0.3448	0.0615	0.039*	
C10A	0.0762 (2)	0.3039 (2)	−0.11224 (18)	0.0343 (6)	
H10A	−0.0043	0.3216	−0.1470	0.041*	
C11A	0.2074 (2)	0.2658 (2)	−0.18536 (18)	0.0299 (5)	
H11A	0.2176	0.2581	−0.2704	0.036*	
C12A	0.3237 (2)	0.2389 (2)	−0.13428 (16)	0.0287 (5)	
H12A	0.4142	0.2135	−0.1846	0.034*	
C13A	0.3098 (2)	0.2488 (2)	−0.01033 (17)	0.0262 (5)	
H13A	0.3902	0.2291	0.0243	0.031*	
C14A	0.1435 (2)	0.37892 (19)	0.43984 (16)	0.0193 (4)	
O15A	0.22885 (13)	0.43274 (13)	0.36935 (10)	0.0224 (3)	
O16A	0.11716 (14)	0.40479 (13)	0.55831 (10)	0.0222 (3)	
H16A	0.1700	0.4553	0.5738	0.033*	

### Atomic displacement parameters (Å^2^)

**Table:**

	*U*^11^	*U*^22^	*U*^33^	*U*^12^	*U*^13^	*U*^23^
F1B	0.0345 (7)	0.0296 (7)	0.0280 (6)	−0.0177 (6)	−0.0080 (5)	−0.0036 (5)
C1B	0.0189 (11)	0.0165 (10)	0.0214 (10)	−0.0035 (9)	−0.0060 (8)	0.0011 (8)
C2B	0.0186 (11)	0.0170 (10)	0.0188 (10)	−0.0037 (9)	−0.0049 (8)	0.0005 (8)
C3B	0.0239 (12)	0.0185 (11)	0.0193 (10)	−0.0007 (9)	−0.0055 (9)	−0.0032 (8)
C4B	0.0202 (12)	0.0226 (11)	0.0200 (10)	−0.0040 (9)	−0.0021 (9)	0.0001 (8)
C5B	0.0210 (12)	0.0177 (11)	0.0255 (11)	−0.0058 (9)	−0.0077 (9)	−0.0019 (8)
C6B	0.0260 (12)	0.0181 (11)	0.0208 (10)	−0.0027 (9)	−0.0075 (9)	−0.0027 (8)
N7B	0.0285 (10)	0.0287 (10)	0.0176 (8)	−0.0142 (8)	−0.0076 (7)	−0.0002 (7)
C8B	0.0201 (12)	0.0273 (12)	0.0190 (10)	−0.0069 (9)	−0.0056 (9)	−0.0038 (9)
C9B	0.0256 (13)	0.0290 (12)	0.0212 (11)	−0.0030 (10)	−0.0059 (9)	0.0002 (9)
C10B	0.0301 (13)	0.0293 (13)	0.0246 (11)	−0.0065 (10)	−0.0038 (10)	−0.0078 (9)
C11B	0.0337 (14)	0.0421 (14)	0.0163 (10)	−0.0141 (11)	−0.0040 (9)	−0.0042 (10)
C12B	0.0397 (15)	0.0325 (13)	0.0218 (11)	−0.0142 (11)	−0.0054 (10)	0.0041 (9)
C13B	0.0323 (13)	0.0231 (11)	0.0246 (11)	−0.0099 (10)	−0.0051 (9)	−0.0015 (9)
C14B	0.0209 (12)	0.0179 (11)	0.0187 (10)	0.0001 (9)	−0.0080 (9)	−0.0030 (8)
O15B	0.0250 (8)	0.0232 (8)	0.0187 (7)	−0.0093 (6)	−0.0048 (6)	0.0007 (6)
O16B	0.0270 (9)	0.0267 (8)	0.0181 (7)	−0.0109 (6)	−0.0053 (6)	−0.0029 (6)
F1A	0.0329 (7)	0.0277 (7)	0.0367 (7)	−0.0150 (6)	−0.0069 (6)	−0.0049 (5)
C1A	0.0179 (12)	0.0210 (11)	0.0237 (11)	−0.0058 (9)	−0.0021 (9)	−0.0013 (8)
C2A	0.0166 (11)	0.0177 (11)	0.0205 (10)	−0.0010 (8)	−0.0045 (8)	−0.0025 (8)
C3A	0.0188 (12)	0.0204 (11)	0.0231 (10)	−0.0022 (9)	−0.0040 (9)	−0.0002 (8)
C4A	0.0204 (12)	0.0218 (11)	0.0264 (11)	−0.0057 (9)	−0.0023 (9)	0.0039 (9)
C5A	0.0222 (12)	0.0163 (11)	0.0324 (12)	−0.0070 (9)	−0.0076 (9)	0.0001 (9)
C6A	0.0226 (12)	0.0206 (11)	0.0257 (11)	−0.0034 (9)	−0.0081 (9)	−0.0054 (9)
N7A	0.0317 (11)	0.0361 (11)	0.0184 (9)	−0.0189 (9)	−0.0040 (8)	−0.0048 (7)
C8A	0.0291 (13)	0.0243 (12)	0.0203 (10)	−0.0124 (10)	−0.0074 (9)	−0.0021 (8)
C9A	0.0263 (13)	0.0436 (14)	0.0264 (12)	−0.0070 (11)	−0.0029 (10)	−0.0046 (10)
C10A	0.0332 (14)	0.0470 (15)	0.0279 (12)	−0.0103 (11)	−0.0158 (11)	−0.0004 (10)
C11A	0.0392 (15)	0.0322 (13)	0.0211 (11)	−0.0120 (11)	−0.0076 (10)	−0.0006 (9)
C12A	0.0306 (13)	0.0318 (13)	0.0223 (11)	−0.0058 (10)	−0.0022 (10)	−0.0011 (9)
C13A	0.0297 (13)	0.0293 (12)	0.0225 (11)	−0.0086 (10)	−0.0092 (10)	0.0020 (9)
C14A	0.0187 (12)	0.0189 (11)	0.0198 (10)	−0.0013 (9)	−0.0049 (9)	−0.0011 (8)
O15A	0.0254 (8)	0.0252 (8)	0.0187 (7)	−0.0105 (6)	−0.0040 (6)	−0.0008 (6)
O16A	0.0252 (9)	0.0245 (8)	0.0190 (7)	−0.0094 (6)	−0.0055 (6)	−0.0029 (6)

### Geometric parameters (Å, º)

**Table:**

F1B—C5B	1.359 (2)	F1A—C5A	1.357 (2)
C1B—N7B	1.372 (2)	C1A—N7A	1.368 (2)
C1B—C6B	1.407 (3)	C1A—C6A	1.408 (3)
C1B—C2B	1.423 (2)	C1A—C2A	1.428 (2)
C2B—C3B	1.397 (3)	C2A—C3A	1.401 (3)
C2B—C14B	1.468 (3)	C2A—C14A	1.463 (3)
C3B—C4B	1.377 (3)	C3A—C4A	1.375 (3)
C3B—H3B	0.9500	C3A—H3A	0.9500
C4B—C5B	1.390 (2)	C4A—C5A	1.387 (3)
C4B—H4B	0.9500	C4A—H4A	0.9500
C5B—C6B	1.363 (3)	C5A—C6A	1.357 (3)
C6B—H6B	0.9500	C6A—H6A	0.9500
N7B—C8B	1.421 (2)	N7A—C8A	1.424 (2)
N7B—H7B	0.8800	N7A—H7A	0.8800
C8B—C13B	1.382 (3)	C8A—C9A	1.384 (3)
C8B—C9B	1.398 (3)	C8A—C13A	1.388 (3)
C9B—C10B	1.388 (2)	C9A—C10A	1.386 (3)
C9B—H9B	0.9500	C9A—H9A	0.9500
C10B—C11B	1.380 (3)	C10A—C11A	1.380 (3)
C10B—H10B	0.9500	C10A—H10A	0.9500
C11B—C12B	1.381 (3)	C11A—C12A	1.380 (3)
C11B—H11B	0.9500	C11A—H11A	0.9500
C12B—C13B	1.384 (2)	C12A—C13A	1.385 (2)
C12B—H12B	0.9500	C12A—H12A	0.9500
C13B—H13B	0.9500	C13A—H13A	0.9500
C14B—O15B	1.239 (2)	C14A—O15A	1.239 (2)
C14B—O16B	1.3281 (19)	C14A—O16A	1.3315 (19)
O16B—H16B	0.8400	O16A—H16A	0.8400
			
N7B—C1B—C6B	121.45 (17)	N7A—C1A—C6A	121.31 (17)
N7B—C1B—C2B	120.72 (17)	N7A—C1A—C2A	121.16 (17)
C6B—C1B—C2B	117.82 (17)	C6A—C1A—C2A	117.50 (17)
C3B—C2B—C1B	119.53 (17)	C3A—C2A—C1A	119.52 (18)
C3B—C2B—C14B	118.85 (16)	C3A—C2A—C14A	119.23 (17)
C1B—C2B—C14B	121.61 (17)	C1A—C2A—C14A	121.25 (17)
C4B—C3B—C2B	122.73 (17)	C4A—C3A—C2A	122.23 (18)
C4B—C3B—H3B	118.6	C4A—C3A—H3A	118.9
C2B—C3B—H3B	118.6	C2A—C3A—H3A	118.9
C3B—C4B—C5B	115.89 (18)	C3A—C4A—C5A	116.65 (18)
C3B—C4B—H4B	122.1	C3A—C4A—H4A	121.7
C5B—C4B—H4B	122.1	C5A—C4A—H4A	121.7
F1B—C5B—C6B	117.37 (16)	F1A—C5A—C6A	117.90 (17)
F1B—C5B—C4B	117.99 (17)	F1A—C5A—C4A	117.91 (17)
C6B—C5B—C4B	124.64 (19)	C6A—C5A—C4A	124.19 (19)
C5B—C6B—C1B	119.37 (17)	C5A—C6A—C1A	119.90 (18)
C5B—C6B—H6B	120.3	C5A—C6A—H6A	120.1
C1B—C6B—H6B	120.3	C1A—C6A—H6A	120.1
C1B—N7B—C8B	126.58 (16)	C1A—N7A—C8A	126.76 (16)
C1B—N7B—H7B	116.7	C1A—N7A—H7A	116.6
C8B—N7B—H7B	116.7	C8A—N7A—H7A	116.6
C13B—C8B—C9B	119.77 (17)	C9A—C8A—C13A	119.78 (18)
C13B—C8B—N7B	118.66 (17)	C9A—C8A—N7A	121.23 (18)
C9B—C8B—N7B	121.56 (17)	C13A—C8A—N7A	118.94 (17)
C10B—C9B—C8B	119.15 (18)	C8A—C9A—C10A	120.0 (2)
C10B—C9B—H9B	120.4	C8A—C9A—H9A	120.0
C8B—C9B—H9B	120.4	C10A—C9A—H9A	120.0
C11B—C10B—C9B	121.00 (19)	C11A—C10A—C9A	120.3 (2)
C11B—C10B—H10B	119.5	C11A—C10A—H10A	119.9
C9B—C10B—H10B	119.5	C9A—C10A—H10A	119.9
C10B—C11B—C12B	119.33 (18)	C10A—C11A—C12A	119.62 (19)
C10B—C11B—H11B	120.3	C10A—C11A—H11A	120.2
C12B—C11B—H11B	120.3	C12A—C11A—H11A	120.2
C11B—C12B—C13B	120.53 (19)	C11A—C12A—C13A	120.6 (2)
C11B—C12B—H12B	119.7	C11A—C12A—H12A	119.7
C13B—C12B—H12B	119.7	C13A—C12A—H12A	119.7
C8B—C13B—C12B	120.17 (18)	C12A—C13A—C8A	119.67 (19)
C8B—C13B—H13B	119.9	C12A—C13A—H13A	120.2
C12B—C13B—H13B	119.9	C8A—C13A—H13A	120.2
O15B—C14B—O16B	121.64 (17)	O15A—C14A—O16A	121.23 (17)
O15B—C14B—C2B	124.45 (16)	O15A—C14A—C2A	124.38 (16)
O16B—C14B—C2B	113.91 (16)	O16A—C14A—C2A	114.38 (16)
C14B—O16B—H16B	109.5	C14A—O16A—H16A	109.5
			
N7B—C1B—C2B—C3B	177.69 (17)	N7A—C1A—C2A—C3A	−178.56 (18)
C6B—C1B—C2B—C3B	−1.2 (3)	C6A—C1A—C2A—C3A	−0.6 (3)
N7B—C1B—C2B—C14B	−1.0 (3)	N7A—C1A—C2A—C14A	1.4 (3)
C6B—C1B—C2B—C14B	−179.85 (17)	C6A—C1A—C2A—C14A	179.35 (17)
C1B—C2B—C3B—C4B	0.9 (3)	C1A—C2A—C3A—C4A	0.4 (3)
C14B—C2B—C3B—C4B	179.66 (17)	C14A—C2A—C3A—C4A	−179.57 (17)
C2B—C3B—C4B—C5B	0.0 (3)	C2A—C3A—C4A—C5A	0.4 (3)
C3B—C4B—C5B—F1B	179.21 (16)	C3A—C4A—C5A—F1A	178.27 (16)
C3B—C4B—C5B—C6B	−0.8 (3)	C3A—C4A—C5A—C6A	−1.0 (3)
F1B—C5B—C6B—C1B	−179.47 (16)	F1A—C5A—C6A—C1A	−178.49 (16)
C4B—C5B—C6B—C1B	0.6 (3)	C4A—C5A—C6A—C1A	0.8 (3)
N7B—C1B—C6B—C5B	−178.39 (18)	N7A—C1A—C6A—C5A	178.01 (18)
C2B—C1B—C6B—C5B	0.5 (3)	C2A—C1A—C6A—C5A	0.0 (3)
C6B—C1B—N7B—C8B	3.0 (3)	C6A—C1A—N7A—C8A	9.0 (3)
C2B—C1B—N7B—C8B	−175.79 (17)	C2A—C1A—N7A—C8A	−173.10 (18)
C1B—N7B—C8B—C13B	126.8 (2)	C1A—N7A—C8A—C9A	50.9 (3)
C1B—N7B—C8B—C9B	−54.3 (3)	C1A—N7A—C8A—C13A	−131.7 (2)
C13B—C8B—C9B—C10B	−2.5 (3)	C13A—C8A—C9A—C10A	1.4 (3)
N7B—C8B—C9B—C10B	178.62 (18)	N7A—C8A—C9A—C10A	178.73 (18)
C8B—C9B—C10B—C11B	1.9 (3)	C8A—C9A—C10A—C11A	−1.6 (3)
C9B—C10B—C11B—C12B	0.0 (3)	C9A—C10A—C11A—C12A	0.6 (3)
C10B—C11B—C12B—C13B	−1.3 (3)	C10A—C11A—C12A—C13A	0.6 (3)
C9B—C8B—C13B—C12B	1.3 (3)	C11A—C12A—C13A—C8A	−0.7 (3)
N7B—C8B—C13B—C12B	−179.79 (18)	C9A—C8A—C13A—C12A	−0.2 (3)
C11B—C12B—C13B—C8B	0.6 (3)	N7A—C8A—C13A—C12A	−177.65 (17)
C3B—C2B—C14B—O15B	−175.23 (18)	C3A—C2A—C14A—O15A	177.69 (18)
C1B—C2B—C14B—O15B	3.5 (3)	C1A—C2A—C14A—O15A	−2.2 (3)
C3B—C2B—C14B—O16B	4.6 (3)	C3A—C2A—C14A—O16A	−2.4 (3)
C1B—C2B—C14B—O16B	−176.67 (16)	C1A—C2A—C14A—O16A	177.70 (16)

### Hydrogen-bond geometry (Å, º)

**Table:**

*D*—H···*A*	*D*—H	H···*A*	*D*···*A*	*D*—H···*A*
N7*A*—H7*A*···O15*A*	0.88	1.98	2.673 (2)	135
N7*B*—H7*B*···O15*B*	0.88	1.98	2.6770 (19)	136
O16*A*—H16*A*···O15*B*^i^	0.84	1.80	2.6413 (19)	176
O16*B*—H16*B*···O15*A*^i^	0.84	1.77	2.6099 (19)	174
C4*A*—H4*A*···F1*B*^ii^	0.95	2.48	3.426 (2)	174
C4*B*—H4*B*···F1*A*^ii^	0.95	2.52	3.464 (2)	173

Symmetry codes: (i) −*x*+1, −*y*+1, −*z*+1; (ii) −*x*, −*y*, −*z*+1.
